# Development of a Sensitive Chemiluminescence Immunoassay for the Quantification of Folic Acid in Human Serum

**DOI:** 10.1155/2019/5402903

**Published:** 2019-05-21

**Authors:** Xiang Chen, Qiyang Zhou, Ting Zhang, ChunXin Wang, Zheng Yu, Hadji Ahamada, Zhonghu Bai, Xuan Huang

**Affiliations:** ^1^School of Biotechnology, Jiangnan University, Wuxi, China; ^2^National Engineering Laboratory for Cereal Fermentation Technology, Jiangnan University, Wuxi, China; ^3^Jiangsu Testing and Inspection Institute for Medical Devices, Nanjing, China; ^4^The Affiliated Wuxi Maternity and Child Health Care Hospital of Nanjing Medical University, Wuxi, China; ^5^Medical Laboratory, Wuxi People Hospital Affiliated to Nanjing Medical University, Wuxi, China; ^6^Hematology and Clinical Biochemistry Department, Hospital EL-Maarouf, Moroni, Comoros; ^7^The Key Laboratory of Carbohydrate Chemistry and Biotechnology, Ministry of Education, School of Biotechnology, Jiangnan University, Wuxi 214122, China; ^8^Department of Laboratory Medicine, Affiliated Hospital of Jiangnan University, Wuxi, China

## Abstract

Folic acid (FA) is an important vitamin for human growth, especially for pregnant women. FA deficiency is associated with megaloblastic anemia, neural tube defects, cardiovascular diseases, irritability, diarrhea, and psychiatric disorders. Normally, FA molecules bind to folate-binding protein (FBP) in the serum as complex. Before quantify the FA concentration, a releasing procedure should be conducted. Alkaline condition and tris(2-carboxyethyl)phosphine (TCEP) are used to release binding FA to freeing state. In this work, a chemiluminescence immunoassay (CLIA) for human serum FA was established by competition model. Streptavidin (SA) was labeled to magnetic beads by an 1-ethyl-3-(3-dimethylaminopropyl) carbodiimide/N-hydroxysuccinimide (EDAC/NHS) method. Activated biotin molecules were labeled to FBP molecules purified from milk. FA was labeled to horseradish peroxidase (HRP) by EDAC to activate the FA molecules. The pretreated samples or standards were added into the reaction tube with biotin-FBP and FA-horseradish peroxidase (HRP), FA in the sample compete with FA-HRP for binding to biotin-FBP, the signal is inversely proportional to the FA concentration. The method established shows good thermostability and performance. The limitation of detection (LOD) is 0.44 ng/mL. The intra-assay coefficient of variation (CV) is 3.6%–7.1%, the interassay CV is 4.2%–7.5%, and the recovery rate is 92.1%–103.5%. Cross reactivity (CR) was remarkably low with aminopterin, folinic acid, and methotrexate. The method shows good correlation with the FA CLIA product from Beckman Coulter; the equation is *y* = 0.9618*x*−0.1434 while the *R*^2^ value is 0.9224. The established method is sensitive, rapid, and accurate which can fully satisfy for the clinical requirement.

## 1. Introduction

Folate is the generic term for all B-vitamins that exhibit vitamin activity similar to that of folic acid. The various compounds of this class are designated as “folates”. Folic acid is the most oxidised and most stable form of folate. It is an organic compound necessary for the maintenance of normal body processes, synthesis of proteins and nucleic acids, metabolism of amino acids [1, 2], and division of cells. Folate deficiency is associated with megaloblastic anemia, neural tube defects, cardiovascular diseases, irritability, diarrhea, and psychiatric disorders [3–7]. In pregnant women, it may result in neural tube defects, developmental retardation, and early spontaneous abortion [8, 9]. Therefore, in vitro diagnostic of FA is particularly important.

The most common conventional methods for the analysis of FA is microbiological method and radiological immunoassay which are time consuming, labor intensive, or harmful to the environment and human health. High-performance liquid chromatography spectrometry is accurate for FA detection and can be used for the simultaneous detection of several vitamins [10]. However, this method is quite expensive and time consuming.

The CLIA is a simple, sensitive, and cheap method for the high-throughput quantification of analyses in samples [11]. It is widely used nowadays. In this study, a direct competitive immunoassay was developed for the detection of serum FA while FBP is extracted from cow's milk and the best FA/HRP ratio conjugates was prepared. FBP are proteins that specifically bind to folate, typically the folate receptors [12], as the most important and widely used material during the in vivo diagnostic. Its specificity and cross reactivity directly influence the performance of the method developed. FA-HRP conjugate is also one of the most important materials in the immunoassay, different FA/HRP ratio conjugates express different compete abilities.

## 2. Materials and Methods

### 2.1. Reagents and Materials

Dynabeads MyOne™ carboxylic acid beads, EDAC, and NHS are purchased from Sigma; EZ-Link™ Sulfo-NHS-LC-Biotinylation Kit and HABA solution are obtained from Thermo Fisher; EAH-Sepharose 4B and Akta purifier are purchased from GE healthcare; FBP is obtained from Scripps; HRP is purchased from BBI solutions; Streptavidin is purchased from NeuroPeptide from China; Microscope are purchased from Olympus; Auto microplate chemiluminescent analyzer is supplied by Baiming biotechnology from China; and Auto magnetic beads chemiluminescent analyzer is supplied by Zecheng biotechnology from China.

### 2.2. Streptavidin-Coated Magnetic Beads Preparation

We washed the carboxylic acid beads twice with 25 mM MES, pH 6.0 buffer. We added EDAC and NHS solutions to the washed Dynabeads. It was mixed well and incubated with slow tilt rotation at room temperature for 30 min. We also washed the beads twice to remove the supernatant. The required amount of streptavidin was added into the activated magnetic beads and incubated for 30 min at room temperature with slow tilt rotation. We washed the beads twice again and then suspended the beads to 1 mg/mL with a PBS buffer containing 0.5% BSA, 0.05% polysorbate 20, and 0.02% sodium azide.

### 2.3. FBP Isolation and Biotinylated Conjugate Preparation

#### 2.3.1. Isolation

FBP was isolated from fresh milk by affinity chromatography using EAH-Sepharose 4B [13]. Folic acid was coupled to the matrix. The pH of the pretreated fresh milk which was centrifuged was lowered to 3.0 by slowly adding 5 M HCl under agitation to dissociate the bounded folate from FBP. The sepharose gel was added to the solution above, and the pH was increased to 7.0 by slowly adding 5 M NaOH. After hours of agitation, the sepharose gel was washed on a glass filter with deionized water until a clear elute was obtained. Then the gel was packed into a column and connected to Akta system. The impurity was washed with 0.1 M phosphate buffer, containing 0.5 M NaCl, pH 7.2. The bound FBP was dissociated and eluted by 0.1 M acetate buffer, pH 3.5. The collected FBP containing fraction was dialysed against 0.1 M phosphate buffer, containing 0.5 M NaCl, pH 7.2 before the biotinylated conjugate preparation.

#### 2.3.2. Conjugate Preparation

Dissolve EZ-Link Sulfo-NHS-LC-Biotin in 0.1 M phosphate buffer, pH 7.2, and add it into the dialysed FBP solution above. Incubate at room temperature for 60 minutes. Then remove the impurities with a desalination column.

### 2.4. FA-HRP Conjugate Preparation

Dissolve different weight of folic acid and EDAC in separated 0.05 M sodium bicarbonate solutions. Mix them after the solute dissolved completely. Dissolve HRP in 0.1 M phosphate buffer, pH 7.2. Then, add the mixed solution into the HRP solution and lower the pH to 5.8 with diluted hydrochloric acid. Incubate for 5 hours at room temperature. Finally, remove the impurities with a desalination column.

### 2.5. Development and Optimization of the CLIA Method

Normally, FA molecules bind to FBP in the serum as complex. Before quantifying the FA concentration, a releasing procedure should be conducted. Alkaline condition and TCEP are commonly used because of its low toxicity and odorless property compared to *β*-mercaptoethanol. The essential reagents required for the immunoassay include biotin-FBP, enzyme-FA conjugates, and FA. Upon mixing the biotin-FBP with a serum pretreated containing free FA, a reaction results between the FBP and FA. A simultaneous reaction between the biotin-FBP attached to the SA immobilized on the magnetic beads. The enzyme activity is inversely proportional to the FA concentration. By utilizing several different serum references of known antigen concentration, a dose response curve can be generated, and the FA concentration of an unknown can be ascertained. In this study, factors such as FA releasing procedure, FBP concentration, FA-HRP type and concentration, and incubation time were optimized.

### 2.6. Method Performance

#### 2.6.1. Limitation of Detection

20 “zero” standard are detected, and the MEAN-3SD value is calculated, and the corresponding concentration is the limitation of detection.

#### 2.6.2. Precision and Recovery

Three serum samples of different FA concentrations were tested and duplicated separately in one experiment and repeated in 20 days, and the intra-assay and interassay CV are calculated. The concentration of FA solutions was added to three serum samples of different FA concentrations, and the recovery rate is calculated.

#### 2.6.3. Cross Reactivity

The specificity of the FBP used to selected substances was evaluated by adding the interfering substance to a serum matrix at various concentrations. The cross reactivity (CR) is defined at the point where the reduction in signal corresponds to 50% of the signal achieved in the absence of analyte (B/B0 of 50%), as a percentage of the analyte concentration given the same fall in signal. The CR values were calculated as follows [14]:

(1) $$\begin{array}{c} \text{CR}\left(\%\right)=\frac{{\text{IC}}_{50}\text{ of FA}}{{\text{IC}}_{50}\text{ of competitor}}\times 100\%. \end{array}$$

#### 2.6.4. Accelerated Stability

The whole kit including SA-magnetic bead, biotin-FBP, FA-HRP, and FA standards was incubated at 37°C for 7 days compared the signals of standards and concentration of controls at different days.

### 2.7. Methodology Comparison

The CLIA method was compared with a reference chemiluminescence immunoassay method of Beckman Coulter. Biological specimens were used with values that ranged from 1.1 ng/ml–23.9 ng/ml. The total number of such specimens is 240.

## 3. Results

### 3.1. Characterization of SA-Coated Magnetic Beads

**Figure 1:**
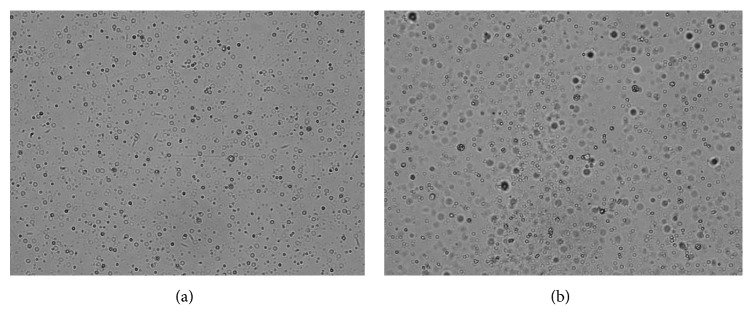
The microphotographs of unlabeled and labeled beads. (a) Unlabeled beads. (b) SA labeled beads.

Add 30 *μ*L SA-coated magnetic beads prepared and 50 *μ*L biotin-HRP (50 ng/mL) into a reaction tube incubated for 30 min at 37°C and wash the beads 3 times with washing buffer. Then add 100 *μ*L signal reagent to detect the signals. The RLUs result is 803,394 which indicates that the SA is successfully labeled onto the surface of the magnetic beads. The SA labeled beads showed only a little aggregation compared to unlabeled beads. The microphotographs are shown in Figure 1.

### 3.2. Characterization of FBP and Biotin-FBP Conjugates

The purified FBP was coated on the surface of the microwells. Add 50 *μ*L FA-HRP conjugates (0.5 *μ*g/mL) prepared in our lab, incubated for 30 min at 37°C, and wash the wells 5 times with washing buffer. Then add 100 *μ*L signal reagent to detect the signals. The RLUs result is 249,403 indicates that the purified FBP have good biological activity. To estimate biotin incorporation, a solution containing the biotinylated FBP is added to a mixture of HABA and avidin solution purchased from Sigma. The biotin/FBP mole ratio is approximately 6.3 calculated by the method descript in the kit manual.

### 3.3. Characterization of FA-HRP Conjugates

**Figure 2:**
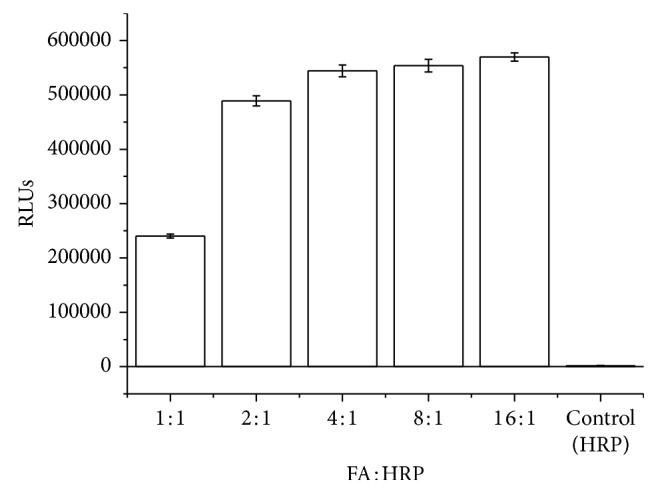
FA-HRP Conjugates verification (*n*=3).

Purchased FBP from Scripps coated microplates were prepared to verify whether FA is labeled to HRP molecules successfully. Add 50 *μ*L different FA-HRP conjugates (1.0 *μ*g/mL) with HRP as the control into the FBP coated wells; then incubate for 30 min at 37°C, and wash the plate 5 times with washing buffer. Then add 100 *μ*L signal reagent. The results indicate that FA was successfully labeled to HRP. See the results in Figure 2.

### 3.4. Development of the CLIA Method

#### 3.4.1. Method Procedure

Add peptide 30 *μ*L serum and 10 *μ*L stabilizer reagent which is used to stabilize the folate in the sample into the reacting tube. Then add 20 *μ*L alkaline solution called releasing reagent into the tube. After a 10 min incubation to release the folate from FBP completely, decrease the pH to neutral with an acid solution called neutralization buffer. Add peptide 50 *μ*L FBP-Biotin into the tube and hold for 20 min, then add 50 *μ*L FA-HRP conjugated, and incubate for 5 min. Add 30 *μ*L magnetic bead coated with SA and incubate for another 5 min. Lastly, the magnetic bead was washed three times with washing buffer, and then add 200 *μ*L signal reagent. The reader will give the signal values. All the incubation temperature should be controlled at 37°C.

#### 3.4.2. Optimization of Biotin-FBP and FA-HRP Concentration

**Figure 3:**
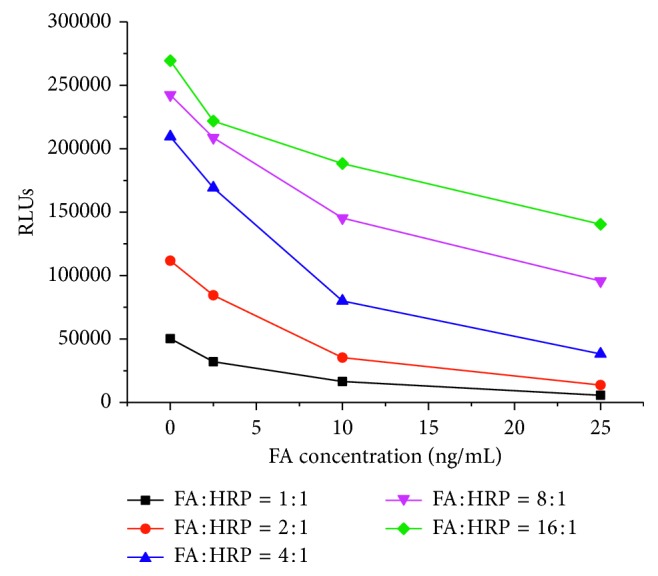
Signals decrease of different FA/HRP ratio conjugates.

Different FA/HRP ratio conjugates present different competing abilities. Signals of standards decrease more of low ratio compared to high ratio. Conjugates labeled by 2 : 1 of FA molar excess shows the biggest Signal (0 ng/mL)/Signal (25 ng/mL) value with relative high signals. The results are shown in Figure 3. A series concentration of biotin-FBP and FA-HRP were investigated, the signals and Signal (0 ng/mL)/Signal (25 ng/mL) value is acceptable when biotin-FBP concentration is 520 ng/mL and FA-HRP concentration is 500 ng/mL. The optimization results are shown in Table 1.

#### 3.4.3. Optimization of Incubation Time

**Figure 4:**
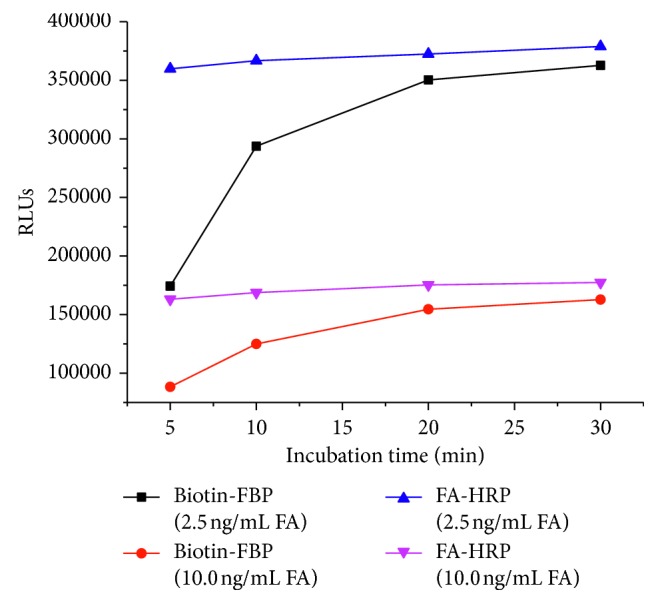
Optimization of the incubation time.

The biotin-FBP and FA-HRP incubation time is studied, respectively. The procedure remains unchanged described in method procedure except the incubation time. The signals increase as the incubation time is lengthened at first, but remain constant while the reaction achieves dynamic equilibrium. For clinical request for saving time, we choose 20 min incubation of biotin-FBP and 5 min incubation of FA-HRP as the best incubation condition which achieve high signals with a relative low variable coefficient. The signal results are shown in Figure 4 and Table 2.

#### 3.4.4. Folic Acid Releasing Procedure Optimization

**Figure 5:**
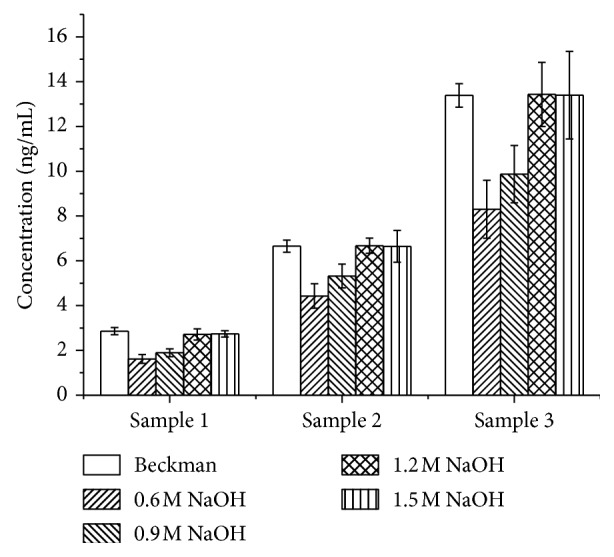
Optimization of the concentration of sodium hydroxide (*n*=3).

**Figure 6:**
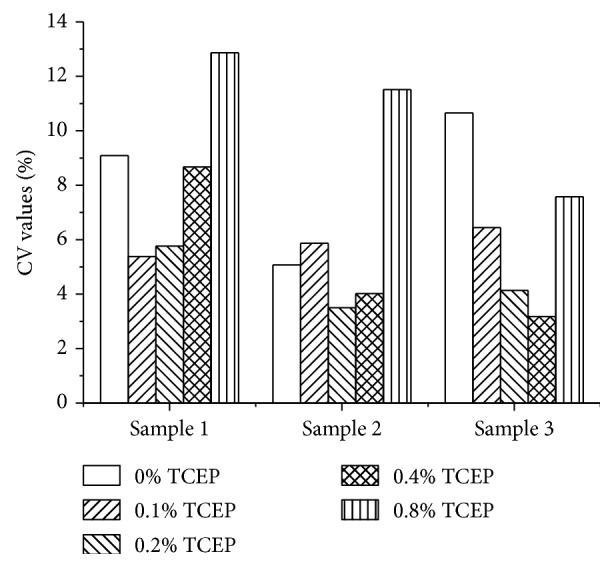
Optimization of the concentration of TCEP (*n*=3).

**Figure 7:**
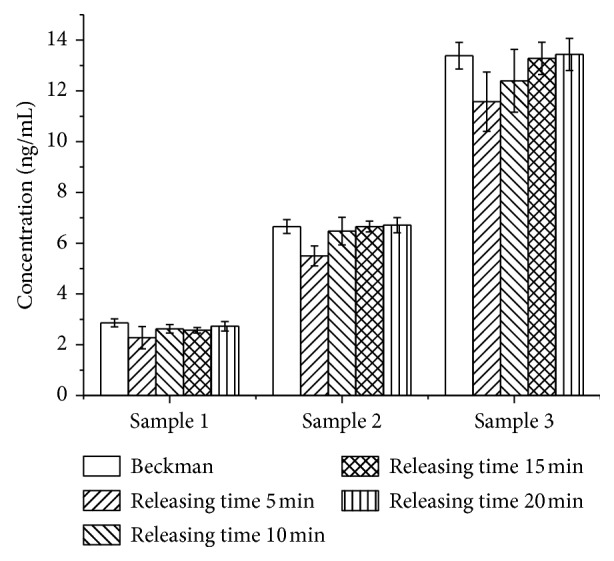
Optimization of the releasing time (*n*=3).

Alkaline condition and TCEP are used in this study to release the binding FA to freeing state. The more alkaline the releasing solution, the more the FA released. According to the results, 1.2 M sodium hydroxide-releasing solution incubated with samples for 15 min at 37°C seems releasing FA completely but with unacceptable CV values. See the results in Figure 5. When adding TCEP into the stabilizing solution, CV values decrease to an acceptable level. However, when adding excessive TCEP higher than 0.2%, it may reduce the activity of HRP conjugates or biotin-FBP, the signals of standards and samples decrease. The results are shown in Figure 6. Therefore, 1.2 M sodium hydroxide-releasing solution and 0.2% TCEP are chosen as the important releasing agents. Further studies were conducted to optimize the releasing time, and 15 min is the best for accuracy and precision did not get better while extension the releasing time, and the results are shown in Figure 7.

#### 3.4.5. Method Performance

**Figure 8:**
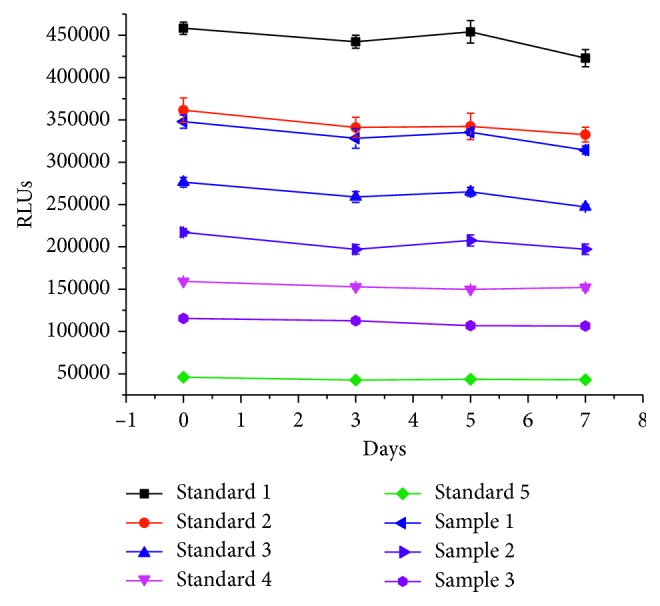
Reagents stability at 37°C.

The standard curve of signal against FA concentration have a regression correlation coefficient (*R*^2^) of 0.999 by 4-parameter logistic function curve fitting, the equation is *Y* = 440081.9 + (−476203.8)/(1 + EXP(−(−2.8074 + 1.3656 ∗ ln (*X*)))). The LOD is 0.44 ng/ml. The intra-assay CV is 3.6%–7.1%, and the interassay CV is 4.2%–7.5%, and the results are shown in Table 3. The recovery rate is 92.1%–103.5%, and the results are shown in Table 4. The developed methods have high selectivity for FA. CR values were less than 0.1% with aminopterin, folinic acid, and methotrexate. The accelerated stability studies show the kit with excellent stability. The signals of the standards and the samples at different days under 37°C incubation change no more than 10% compare to the control, see the results in Figure 8.

### 3.5. Methods Comparison

**Figure 9:**
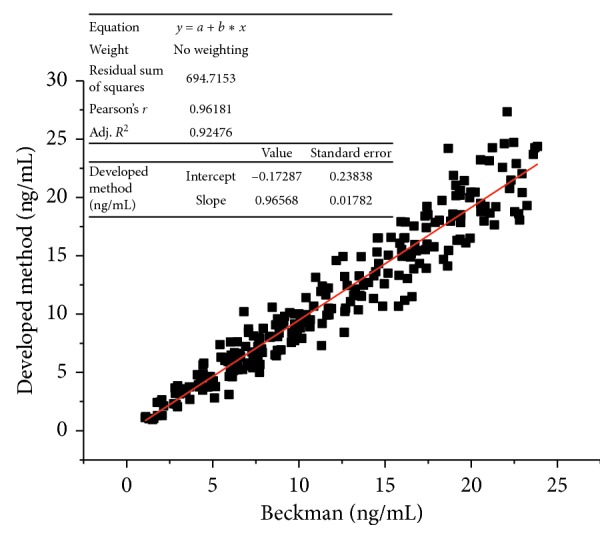
Comparison the developed CLIA method with Beckman Coulter.

In this study, 240 serum samples were tested. The least square regression equation and the correlation coefficient were computed for this FA CLIA method in comparison with the reference method. Only slight amounts of bias between the developed and the reference method are indicated by the closeness of the mean value. The least square regression equation and correlation coefficient indicates excellent method agreement. The results are shown in Figure 9.

## 4. Discussion and Conclusion

According to the previous literatures, there are indeed many quantification methods for FA such as chemiluminescence reaction based on peroxomonosulfate-cobalt (II) system [15], capillary electrophoresis system [16], sodium hypochlorite-folic acid-emicarbazide hydrochloride system [17], flow injection analysis system, [18] and some other methods [19, 20]. Although the LOD is as low as 6 × 10^−10^ mol/L [15], these methods are mainly used for quantification of FA in pharmaceutical preparations, juices, spinach, and human urine which need not release from its binding state as in human serum. HPLC-MS/MS [21] can be an accurate and quick method. But the cost of instruments, consumables, and the low throughput limited the wide application especially in the inspection and organizations with many samples.

This study established a chemiluminescence immunoassay for the quantification of human serum FA with good performance and overall stability. The method comparison shows that the CLIA method established has a good correlation with the imported FA kit from Beckman Coulter which is highly admitted and widely used.

For the competitive CLIA method, the material such as FA-HRP conjugates should be paid more attention as different FA-HRP incorporation show different compete ability, so the label procedure of the conjugates should be strictly controlled to minimize the batch difference. Normally, FA molecules bind to FBP in the serum as complex; the samples should be pretreated to release FA to the freeing state. In this study, 1.2 M sodium hydroxide and 0.2% TCEP were used as releasing agents to maximize the release of FA while keep a low variable coefficient.

During the development of the method, an interesting unusual phenomenon was found. After adding the substrate, the signal of the serum samples increases slowly and reaches the peak at about 1 to 8 min while the standards reach the peak at about 1 min. Mostly, the signals of the samples reach the peak at 1 min, sometimes at 5 or 8 min. Thus, the calculated concentration of the samples varies very much between replicates inter- or intra-assay. This phenomenon did not present in many other projects including VB12, FER, and 25-OH-Vitamin D in our lab. We assume that there are some folate isomers with reducibility which cannot be washed away to interfere the chemiluminescence reaction. So appropriate amount of potassium iodate was added in the incubation buffer to oxidize these substances and minimize the interference. Fortunately, the serum sample signals increase the same as the standards. But further studies should be conducted to investigate the deeper reason.

## Figures and Tables

**Table 1:** Biotin-FBP and FA-HRP concentration optimization.

Biotin-FPB (*μ*g/mL)	0.52			0.26			0.13		
FA-HRP (*μ*g/ml)	1	0.5	0.25	1	0.5	0.25	1	0.5	0.25
Standard 1 (0 ng/mL)	1065638	652382	224155	633363	353217	129995	417738	217887	79858
Standard 2 (2.5 ng/mL)	980613	537333	208843	509083	288071	98061	319829	137721	52073
Standard 3 (5.0 ng/mL)	687334	439226	167008	377044	198141	70016	208496	113568	30422
Standard 4 (10.0 ng/mL)	556606	325643	102205	240137	117481	40667	111972	55471	16877
Standard 5 (25.0 ng/mL)	258853	121479	41827	84444	34271	8641	24247	9238	1727

**Table 2:** Precision results of the optimized incubation time (*n*=10).

Standards	Mean	SD	CV (%)
2.5 ng/mL	371415	5456	1.47
10.0 ng/mL	162957	4472	2.74

**Table 3:** Intra-and interassay CV.

Samples	Intra-assay CV (*n*=20)			Interassay CV (*n*=20)		
	Mean (ng/mL)	SD	CV (%)	Mean (ng/mL)	SD	CV (%)
1	2.58	0.21	8.1	2.65	0.31	11.7
2	6.49	0.36	5.5	6.31	0.46	7.3
3	12.64	0.59	4.7	13.54	0.59	4.4

**Table 4:** Analytical recovery.

Sample	FA concentration (ng/mL)			
	Added concentration	Tested	Expected	Recovery (%)
1	0	2.83		
	2.50	4.91	5.33	92.1
	5.00	7.44	7.83	95.0
	10.00	13.28	12.83	103.5

2	0	8.58		
	2.50	10.41	11.08	94.0
	5.00	13.71	13.58	101.0
	10.00	17.79	18.58	95.7

## Data Availability

The data used to support the findings of this study are available from the corresponding author upon request.
